# Alignment-Free Analysis of Whole-Genome Sequences From Symbiodiniaceae Reveals Different Phylogenetic Signals in Distinct Regions

**DOI:** 10.3389/fpls.2022.815714

**Published:** 2022-04-26

**Authors:** Rosalyn Lo, Katherine E. Dougan, Yibi Chen, Sarah Shah, Debashish Bhattacharya, Cheong Xin Chan

**Affiliations:** ^1^Australian Centre for Ecogenomics, School of Chemistry and Molecular Biosciences, University of Queensland, Brisbane, QLD, Australia; ^2^Department of Biochemistry and Microbiology, Rutgers University, New Brunswick, NJ, United States

**Keywords:** Symbiodiniaceae, coral symbionts, *k*-mers, alignment-free phylogenetics, phylogeny, symbiosis, genome evolution

## Abstract

Dinoflagellates of the family Symbiodiniaceae are predominantly essential symbionts of corals and other marine organisms. Recent research reveals extensive genome sequence divergence among Symbiodiniaceae taxa and high phylogenetic diversity hidden behind subtly different cell morphologies. Using an alignment-free phylogenetic approach based on sub-sequences of fixed length *k* (i.e. *k*-mers), we assessed the phylogenetic signal among whole-genome sequences from 16 Symbiodiniaceae taxa (including the genera of *Symbiodinium*, *Breviolum*, *Cladocopium*, *Durusdinium* and *Fugacium*) and two strains of *Polarella glacialis* as outgroup. Based on phylogenetic trees inferred from *k*-mers in distinct genomic regions (i.e. repeat-masked genome sequences, protein-coding sequences, introns and repeats) and in protein sequences, the phylogenetic signal associated with protein-coding DNA and the encoded amino acids is largely consistent with the Symbiodiniaceae phylogeny based on established markers, such as large subunit rRNA. The other genome sequences (introns and repeats) exhibit distinct phylogenetic signals, supporting the expected differential evolutionary pressure acting on these regions. Our analysis of conserved core *k*-mers revealed the prevalence of conserved *k*-mers (>95% core 23-mers among all 18 genomes) in annotated repeats and non-genic regions of the genomes. We observed 180 distinct repeat types that are significantly enriched in genomes of the symbiotic versus free-living *Symbiodinium* taxa, suggesting an enhanced activity of transposable elements linked to the symbiotic lifestyle. We provide evidence that representation of alignment-free phylogenies as dynamic networks enhances the ability to generate new hypotheses about genome evolution in Symbiodiniaceae. These results demonstrate the potential of alignment-free phylogenetic methods as a scalable approach for inferring comprehensive, unbiased whole-genome phylogenies of dinoflagellates and more broadly of microbial eukaryotes.

## Introduction

Dinoflagellate algae in the family Symbiodiniaceae are extensively studied because they ‘power’ (through the provision of photosynthates) coral reefs and other marine taxa that often inhabit oligotrophic environments. This family encompasses a broad spectrum of symbiotic associations with varied host specificity (González-Pech et al., 2019) and plays a direct role in coral resilience by conferring differential thermal tolerance and influencing colony growth rate (Baker, 2003; Ortiz et al., 2013; D’Angelo et al., 2015). Some Symbiodiniaceae do not associate with a host and are believed to be free-living. Our understanding of Symbiodiniaceae diversity has long been complicated by their subtly different unicellular morphologies. These taxa were once assumed to be a single panmictic species, colloquially known as ‘zooxanthellae’ or as the single genus ‘*Symbiodinium’*. The more-recent use of molecular markers, in combination with morphological and ecological data, better captured the true diversity of these taxa as the family Symbiodiniaceae (LaJeunesse et al., 2018); of the 15 clades described, 11 to date have been formally described as distinct genera (LaJeunesse et al., 2018, 2021; Nitschke et al., 2020; Pochon and LaJeunesse, 2021). The commonly used molecular markers for genotyping Symbiodiniaceae symbionts include large subunit ribosomal RNAs, the internally transcribed spacer (ITS) regions and plastid 23S rRNAs (Cunning et al., 2017; Hume et al., 2018; LaJeunesse et al., 2018). The concatenated multi-gene approach was also used to assess phylogenetic diversity within this group (Pochon et al., 2014; Pochon et al., 2019). These approaches are based on the implicit assumption that the evolutionary history of the chosen markers faithfully reflects Symbiodiniaceae evolution, against the backdrop of intragenomic variation of ITS2 sequences and potential cryptic species (Thornhill et al., 2007; Stat et al., 2011; Arif et al., 2014; Hume et al., 2019).

The available genome data from Symbiodiniaceae is sufficient to assess their phylogenetic diversity using genome-wide features. Strictly orthologous (single-copy) genes from different taxa are expected to arise *via* speciation events, thus the consensus of their individual evolutionary histories (e.g. gene/protein trees) can be assumed to reflect organismal phylogenetic relationships. This approach was successfully adopted to infer representative species phylogenies (Aguileta et al., 2008; Li et al., 2017), including the dinoflagellate tree of life (Price and Bhattacharya, 2017; Stephens et al., 2018). Extending strictly orthologous genes to include homologous genes, the recent Symbiodiniaceae phylogeny inferred using 28,116 gene families (each containing four or more genes) recovered from 15 dinoflagellate genomes (González-Pech et al., 2021) is congruent to that inferred using the LSU rRNA (LaJeunesse et al., 2018).

However, use of these genes, while reasonable, limits the focus to a small fraction of these massive genomes. The entire exon regions comprise no more than 10% of assembled genome sequences of Symbiodiniaceae (González-Pech et al., 2021); many conserved, lineage-specific genes encode functions that are yet to be uncovered (Stephens et al., 2018). The other genomic regions (e.g. repetitive regions and introns) comprise the majority of genome sequences (Supplementary Table 1) and likely play essential roles in the regulation of gene expression and genome evolution. However, these regions are largely ignored in conventional phylogenetic analyses based on multiple sequence alignment. In addition, multiple sequence alignment is based on the implicit assumption of full-length contiguity of homologous sequences, which is often violated by horizontal genetic transfer, and when aligning whole-genome sequences, genetic rearrangement (Chan and Ragan, 2013).

Alignment-free phylogenetic approaches (Bonham-Carter et al., 2014; Ren et al., 2018; Bernard et al., 2019; Zielezinski et al., 2019; Bernard et al., 2021) provide a scalable alternative to infer phylogenetic relationships from whole-genome sequences: for example, based on the shared similarity of short, sub-sequences of defined length *k* (i.e. *k*-mers). The use of *k*-mers has been previously adopted for identifying putative outliers in high-throughput sequence read data (Mapleson et al., 2017) and to compute local alignment boundaries between two genome sequences (Jain et al., 2018). In a *k*-mer-based phylogenetic approach, the proportion of shared *k*-mers between two genomes is used to calculate a pairwise distance that is used to derive a phylogenetic relationship. This approach is robust, even with the existence of genetic recombination and rearrangements (Chan et al., 2014; Bernard et al., 2016a), and has been shown to efficiently reconstruct biologically relevant phylogenies among hundreds (Bernard et al., 2016b; Jacobus et al., 2021) and thousands of microbial genomes (Bernard et al., 2018). By not focusing on specific genes, alignment-free phylogenetic approaches enable the capture of phylogenomic signal from whole-genome sequences. A recent assessment of Symbiodiniaceae phylogeny using repeat-masked genome data from 15 dinoflagellate taxa (González-Pech et al., 2021) revealed largely consistent results with that found using current systematics approaches (LaJeunesse et al., 2018). However, given the prevalence of repetitive regions in the genomes, the impact of these regions and other genomic regions on the overall phylogenetic signal remains an open question.

Here, using genome data from 18 dinoflagellate taxa (16 from Symbiodiniaceae), we assess the phylogenetic signal captured by *k-*mers from distinct genomic regions and the implications of these results on Symbiodiniaceae evolution. Our results provide novel insights into the use of whole-genome sequences to elucidate Symbiodiniaceae diversification and evolution. A major focus of this study is on the representation of evolutionary relationships as networks that lead to understanding not gained using the conventional representation of tree.

## Materials and Methods

### Genome Data

The 18 annotated genome data sets used in this study are shown in Table 1. Of these, 16 are from Symbiodiniaceae taxa representing five genera: *Symbiodinium* (9; Shoguchi et al., 2018; González-Pech et al., 2021; Nand et al., 2021), *Breviolum* (1; Shoguchi et al., 2013), *Cladocopium* (3; Shoguchi et al., 2018; Robbins et al., 2019), *Durusdinium* (2) and *Fugacium* (1; Li et al., 2020), whereas the remaining two are from the sister lineage *Polarella glacialis* (Stephens et al., 2020) as outgroup. Symbiodiniaceae and *Polarella* are classified in the dinoflagellate Order Suessiales. To maximise taxon representation in our assessment of alignment-free phylogenetic inference, we included three available genome data sets: the assembled genome of *Cladocopium goreaui* revised from Liu et al. (2018) and two assembled genomes from distinct isolates of *Durusdinium trenchii*, CCMP2556 and SCF082 (Dougan et al., 2022). All genome data used in this study are available upon request.

**Table 1:** Genome data of the 18 dinoflagellate taxa (Order Suessiales) used in this study.

Species	Isolate	Lifestyle	Scaffolds	Scaffold N50 length (Kb)	References
*Breviolum minutum*	Mf1.05b.01	Symbiotic	21,898	125.23	Shoguchi et al. (2013)
*Cladocopium goreaui*	SCF055	Symbiotic	6,843	353.90	Revised from Liu et al. (2018)
*Cladocopium* sp.	C15	Symbiotic	34,589	50.69	Robbins et al. (2019)
*Cladocopium* sp.	C92	Symbiotic	6,685	247.56	Shoguchi et al. (2018)
*Durusdinium trenchii*	CCMP2556	Symbiotic	29,137	774.26	Dougan et al. (2022)
*Durusdinium trenchii*	SCF082	Symbiotic	44,682	398.48	Dougan et al. (2022)
*Symbiodinium linucheae*	CCMP2456	Symbiotic	37,772	58.08	González-Pech et al. (2021)
*Symbiodinium microadriaticum*	04-503SCI.03	Symbiotic	57,558	49.98	González-Pech et al. (2021)
*Symbiodinium microadriaticum*	CassKB8	Symbiotic	67,937	42.99	González-Pech et al. (2021)
*Symbiodinium microadriaticum*	CCMP2467	Symbiotic	94	9,963	Nand et al. (2021)
*Symbiodinium tridacnidorum*	CCMP2592	Symbiotic	6,245	651.26	González-Pech et al. (2021)
*Symbiodinium tridacnidorum*	Sh18	Symbiotic	16,175	132.49	Shoguchi et al. (2018)
*Symbiodinium necroappetens*	CCMP2469	Opportunistic	104,583	14.53	González-Pech et al. (2021)
*Symbiodinium natans*	CCMP2548	Free-living	2,855	610.50	González-Pech et al. (2021)
*Symbiodinium pilosum*	CCMP2461	Free-living	48,302	62.44	González-Pech et al. (2021)
*Fugacium* kawagutii	CCMP2468	Free-living?	29,213	13,533	Li et al. (2020)
*Polarella glacialis*	CCMP1383	Free-living, psychrophilic	33,494	170.30	Stephens et al. (2020)
*Polarella glacialis*	CCMP2088	Free-living, psychrophilic	37,768	129.21	Stephens et al. (2020)

*All taxa are classified in family Symbiodiniaceae except the outgroup Polarella glacialis*.

For analysis of whole-genome sequence (WGS) data, the entire assembled genome sequences were used. To generate the repeat-masked whole-genome sequence (rmWGS) data, repetitive elements were deleted from the assembled genome sequences; for this purpose, we used information about annotated repeats from the published genome studies (Table 1) or annotated repeats per the steps described below. The remaining short (<1 Kb) sequences were removed using Seqkit (Shen et al., 2016), yielding the final rmWGS data.

For an in-depth analysis of repeats, we adopted a consistent approach to identify repetitive elements from each genome. To identify repetitive elements, *de novo* repeats were identified in each assembled genome using RepeatModeler v1.0.11 at default setting. Combining these *de novo* repeats with known repeats (RepeatMasker library) as a library, repetitive elements in the genome were identified using RepeatMasker v4.0.7 with options -e ncbi -gff -no_is -a.^1^

The distinct strand-specific genomic regions of coding sequences (CDSs) and introns were extracted from the assembled genomes based on the annotated genome features. The predicted proteins from the CDS regions were used in the analysis of protein sequences. Some predicted CDSs (i.e. the annotated exons) and thus also the introns, particularly tandemly repeated genes, were also annotated as repetitive elements using the approach described above; although regions of CDSs and introns are mutually exclusive, each of these is not mutually exclusive to the annotated repeats. The total bases for each curated data sets for each genome are shown in Supplementary Table 1; the sum of rmWGS and repeats for each genome approximates 100% of the WGS.

### Inference of Reference Phylogeny Using Large Subunit rRNA

Phylogenetic inference using the 28S ribosomal large subunit (LSU) was performed on the D1-D2 LSU region using sequences extracted from the genomes if a full-length sequence from that region was found. For genomes in which the full-length sequence could not be recovered, it was supplemented with a representative LSU obtained from NCBI for either the same species or ITS2 type. The LSU sequences were aligned using MAFFT v7.487 (Katoh and Standley, 2013) at *-linsi* mode followed by phylogenetic inference with IQ-TREE v2.0.5. (Minh et al., 2020) using ultrafast bootstrap (Hoang et al., 2018) for 1,000 replicate samples, that is, parameters -s -bb 1,000. All LSU sequences used in this analysis are available as Supplementary Data 1.

### Optimisation of *k* Length for Phylogenetic Inference

Because the choice of *k* for phylogenetic inference is sensitive to the extent of sequence divergence, we followed the method of Greenfield and Roehm, (2013) and Gonzalez-Pech et al. (2021) to identify the optimal *k* independently for each data set, that is, the *k* value that maximises the proportions of distinct and unique *k*-mers in nucleotide sequences (Supplementary Figure 1). The *k* value identified this way was found to have the greatest distinguishing power for phylogenetic analysis (Greenfield and Roehm, 2013). For each nucleotide data set, Jellyfish v2.3.0 (Marçais and Kingsford, 2011) was used to extract and count *k*-mers (*k* between 11 and 25, step size = 2). For the repeats data set, the proportion of unique *k*-mers appears to approximate the maximum at *k* = 21 (Supplementary Figure 2A), but short *k*-mers are not sufficiently distinct (e.g. proportion of distinct *k*-mers <0.5 for all genomes when *k* ≤ 15; Supplementary Figure 2B). In this instance, we assessed *k* values in a larger range, between 11 and 51 (step size = 2) and inferred the phylogenetic tree of repeats using *k* = 21, 35 and 51 (Supplementary Figure 2C). We chose *k* = 51 as the representative tree topology because this value has the greatest power to distinguish repeats based on the proportions of unique and distinct *k*-mers, and the tree topology shows a clear resolution of the distinct genera of Symbiodiniaceae.

For protein sequences, the determination of optimal *k* is not as straightforward. Jellyfish (Marçais and Kingsford, 2011), designed only for extracting *k*-mers from nucleotide sequences, is not applicable in this instance. An earlier benchmark study (Chan et al., 2014) revealed that shorter *k* is more optimal for phylogenetic analysis of protein sequences (with 20 possible amino acids for each character), compared to nucleotide sequences (with four possible nucleotides for each character), supporting the notion that the optimal *k* for sequence analysis is negatively correlated to the alphabet size (Forêt et al., 2006; Höhl and Ragan, 2007; Forêt et al., 2009). Here, we assessed the appropriate *k* value based on the phylogenetic trees that were individually inferred from distances independently derived at *k* = 3, 5, 7 and 9 (see below). We chose *k* = 9 for the analysis of protein sequences, because the corresponding phylogenetic tree is the most similar to the reference topology (see Supplementary Figure 3).

### Alignment-Free Phylogenetic Inference Using *k*-mers

For each data set, the $D_{2}^{S}$ statistic (Reinert et al., 2009) was calculated for each genome pair using the optimal *k* following the algorithm implemented in jD2Stat (Chan et al., 2014); scripts are available at https://github.com/chanlab-genomics/alignment-free-tools/. Here, the $D_{2}^{S}$ statistic describes the number of shared *k*-mers between two genomes, normalised by the probability of the occurrence of each *k*-mer in the sequences. This statistic was then transformed into a pairwise measure of dissimilarity (i.e. distance, *d*). The resulting pairwise distance matrix was used for phylogenetic inference using *neighbor* in PHYLIP v3.69 (Felsenstein, 2005). For the protein sequences, jD2Stat was used to generate *d* based on $D_{2}^{S}$ statistic at *k* = 3, 5, 7 and 9. A $D_{2}^{S}$ distance matrix derived at each size *k* was used for phylogenetic inference as described above, and the resulting trees were visually inspected to identify the optimal *k* (see above).

To infer a network of phylogenetic relatedness based on *k*-mers, we used the method described in Bernard et al. (2016b, 2018). Briefly, for each genome pair, we transformed *d* into a similarity measure *S*, in which *S* = 10–*d*. The *S* value for each genome pair was used to create a network of relatedness using the D3 JavaScript library for data-driven documents;^2^ a Python script for creating a network from a distance matrix is available at https://github.com/chanlab-genomics/alignment-free-tools/. A node in a network represents a genome, and an edge connecting two nodes represents evidence of shared *k*-mers. The threshold function *t* (Bernard et al., 2016b) was used to visualise the network dynamically, for which only edges with *S ≥ t* are displayed.

### Comparison of Tree Topologies

We used Robinson–Foulds distances (Robinson and Foulds, 1981) to assess topological congruence between each *k*-mer-derived tree and the reference tree inferred from multiple sequence alignment of LSU rRNA sequences. We followed the method of Kupczok et al. (2010), using the normalised Robinson–Foulds distance (we denote hereinafter as *RF*) in our assessment; for a tree containing *N* number of leaves, the Robinson–Foulds distance was normalised by the maximum possible distance between two unrooted trees, 2(*N*–3), yielding *RF*. *RF* between two tree topologies was calculated using *RF.dist* of the R package *phangorn*, where *normalise* and *check.labels* are both TRUE (Schliep, 2011). To better assess the impact of topological differences on phylogenetic relationship at the species level, the differential branching order of multiple isolates of the same species was not considered when two topologies were compared. Two tree topologies are identical at *RF* = 0, and they do not share any bipartitions at *RF* = 1.

### Identification of Core *k*-mers and Their Putative Function

The *k*-mers found in every genome in a target group can be interpreted as core genomic elements that are evolutionarily conserved in all members; we used the method of Bernard et al. (2018) to define these *k*-mers as core *k*-mers. To identify core *k*-mers for a target group, we first identified shared *k*-mers between any two genomes within the target group from the output (i.e. the *dump* files) of Jellyfish (see above), and the overlapping *k*-mers found in all pairwise comparisons represent the core *k*-mers of the target group. Using this approach, we identified core *k*-mers for all 18 genomes (i.e. core *k*-mers of Suessiales). To further assess conserved *k*-mers relevant to symbiosis, and given the extensive genome sequence divergence among Symbiodiniaceae (González-Pech et al., 2021), we narrowed our focus and identified core *k*-mers among genomes of symbiotic taxa within the genus *Symbiodinium*: *S. microadriaticum*, *S. tridacnidorum*, *S. linucheae* and *S. necroappetens*.

The core *k*-mers were linked to annotated structural features (e.g. repeats or genes) based on their locations in the genome sequences, using the genome annotation for *S. linucheae* as reference. Locations of *k*-mers were identified using a pblat (Kent, 2002; Wang and Kong, 2019) search against the genome sequences of *S. linucheae*. Strand-specific overlaps of *k*-mers with gene and repeat features were identified using *intersectBed* (−wao -loj -s) implemented in Bedtools suite v2.28.0 (Quinlan and Hall, 2010). The *k*-mers that overlap non-annotated genome regions were considered as ‘unclassified’. An independent analysis using the genome annotation of *S. tridacnidorum* as reference was conducted to verify the consistency of these results. Functional annotation of predicted proteins in *S. linucheae* (González-Pech et al., 2021) was based on the top hit in a BLASTP search (*E  ≤* 10^−5^, minimum query or target cover of 50%) against the UniProt database (Swiss-Prot and TrEMBL). We used the method of Stephens et al. (2018) to define dark genes as those that lack UniProt hits in a BLASTP analysis: that is, they encode a function that is yet to be discovered.

### Prevalence of Repeats in Symbiotic Symbiodinium Versus Free-Living Symbiodinium

To assess the prevalence of repeats in symbiotic lineages, we focused on 825 distinct repeat types that are annotated in all seven genomes of symbiotic *Symbiodinium*. We performed a *t*-test comparing the per-genome sequence proportion and Kimura distances (divergence) of these 825 repeat types between: (a) genomes of symbiotic taxa (i.e. 7 genomes of *S. microadriaticum*, *S. tridacnidorum*, *S. linucheae* and *S. necroappetens*) and (b) genomes of free-living taxa (genomes of *S. pilosum* and *S. natans*). Normality was checked using the Shapiro test and those that violated normality assumptions were log-transformed. Equal variance was assessed using Levene’s test and in the case of unequal variance, a two-sided Welch’s *t*-test for unequal variance was used instead of a two-sided Student’s *t*-test. An adjusted value of *p* ≤0.05 is considered statistically significant. Repeat types that are significantly overrepresented or those with sequences that are significantly more conserved (i.e. significantly lower Kimura distances) were considered more prevalent and evolutionarily conserved in the symbiotic lineages compared to the two free-living lineages.

## Results and Discussion

### Phylogenetic Signal in Distinct Genomic Regions Captured Using *k*-mers

**Figure 1:**
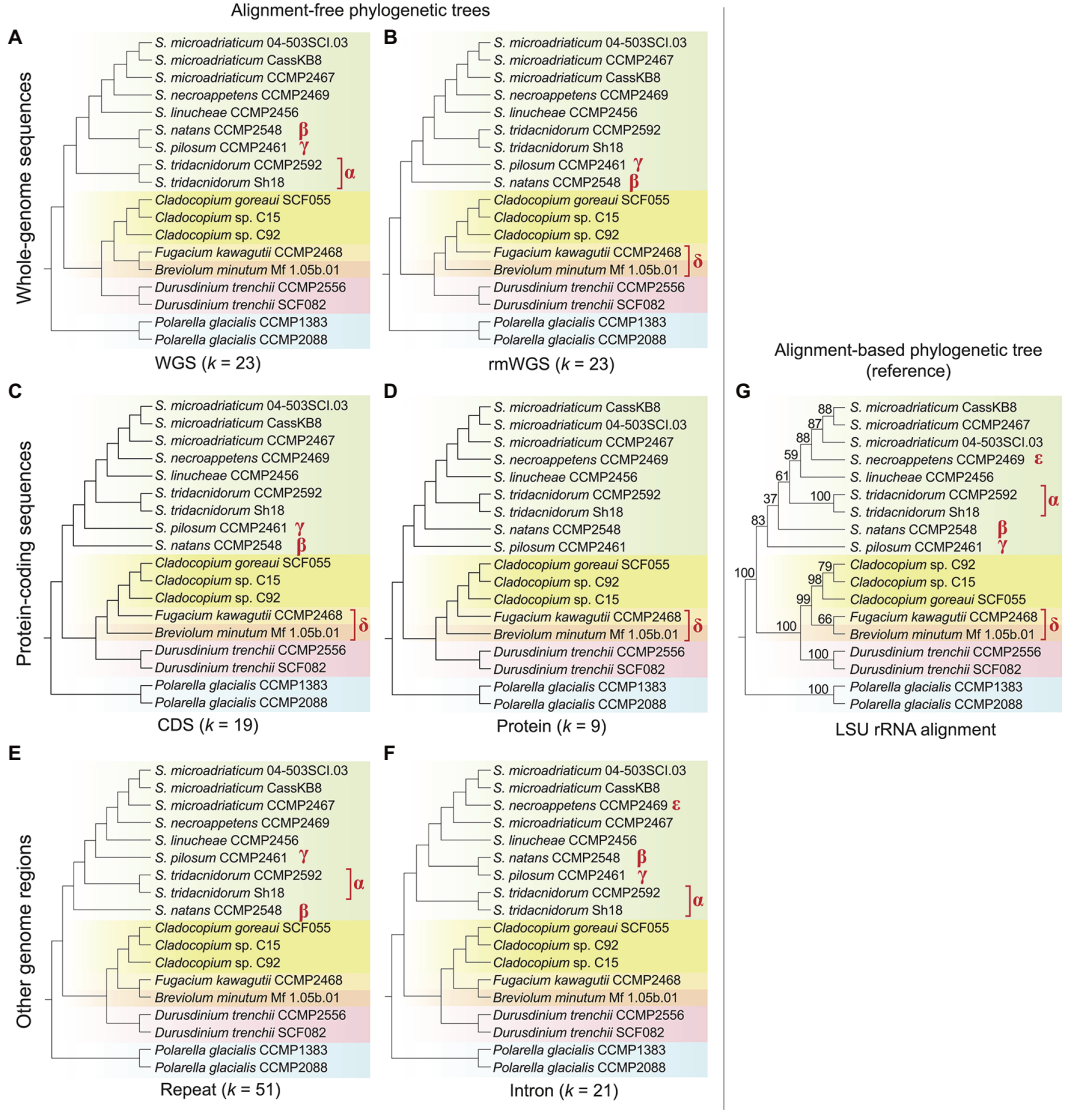
Alignment-free phylogenetic trees of 18 dinoflagellate taxa inferred from: **(A)** whole-genome sequences (WGS), **(B)** repeat-masked whole-genome sequences (rmWGS), **(C)** protein-coding sequences (CDS), **(D)** the encoded protein sequences, **(E)** the annotated repeats and **(F)** the intronic regions. The corresponding *k* used for each inference is shown at the bottom of each tree. **(G)** The topology of reference tree inferred from multiple sequence alignment of LSU rRNA, showing ultrafast bootstrap support (from 1,000 sample replicates). All tree topologies are rooted with *Polarella glacialis* as the outgroup. Differing branching order between each alignment-free tree and the reference tree is indicated with the symbols *α*, *β*, *γ*, *δ* and *ε*. Phylogenetic networks of these alignment-free trees are available as Supplementary Data 2.

Using an alignment-free approach based on *k*-mers, we inferred phylogenetic trees using genome data from 18 dinoflagellate taxa, of which 16 are from the family Symbiodiniaceae (Table 1; see Materials and Methods for detail). Figure 1 shows the tree topologies inferred from each genomic region: whole-genome sequence (WGS) data (Figure 1A), repeat-masked WGS data (rmWGS; Figure 1B), the coding sequences (CDS; Figure 1C), the encoded proteins (Figure 1D), the annotated repeats (Figure 1E) and the introns (Figure 1F). We compared each of these trees against the tree inferred from the multiple sequence alignment of LSU rRNA sequences (Figure 1G) as reference. To account for the distinct properties associated with each genomic region, the choice of *k* was optimised independently for each genome feature (Supplementary Figures 1–3). Branch length information is not shown in Figure 1; this information on alignment-free trees is not readily interpretable and not comparable to the conventional interpretation of number of substitutions per site (Bernard et al., 2016a). The symbols *α*, *β*, *γ*, *δ* and *ε* in Figure 1 denote species-level differences in *k*-mer-inferred lineage positions when compared to the reference tree.

The LSU rRNA-based reference tree topology (Figure 1G; Supplementary Figure 4) largely agrees with the phylogenetic relationships described in LaJeunesse et al. (2018), except for the positions of *Breviolum minutum* and *Fugacium kawagutii* in an independent, weakly supported (B + F) clade (bootstrap support = 66%) external to *Cladocopium*. In the existing phylogeny also based on LSU sequences but from a larger number of taxa (LaJeunesse et al., 2018), *Cladocopium* forms a monophyletic clade with *Fugacium*, and this C + F clade is sister to *Breviolum*. However, the C + F clade in the earlier tree was not robustly supported (bootstrap support = 66%, Bayesian posterior probability = 0.59), thus our observation in Figure 1G is not surprising and likely reflects the smaller number of sequences used in the alignment.

Topological congruence between each *k*-mer-based and the reference tree was quantified using the normalised Robinson–Foulds distance, *RF* (see Materials and Methods); *RF* = 0 indicates complete congruence between the two tree topologies. Overall, all trees inferred using *k*-mers observed in distinct genomic regions are largely congruent (*RF* < 0.3) with the reference. The topology of the protein tree (Figure 1D) is the most similar to the reference (*RF* = 0.13), followed by the WGS (Figure 1A), rmWGS (Figure 1B), CDS (Figure 1C) and repeats (Figure 1E), all with *RF* = 0.20. The intron tree (Figure 1F) has the highest *RF* at 0.27. In protein-coding sequence-based trees (Figures 1C,D), *Breviolum* is sister to the monophyletic clade containing *Cladocopium* and *Fugacium*, as suggested in the existing classification (LaJeunesse et al., 2018). However, in trees inferred from WGS (Figure 1A), introns (Figure 1F) and repeats (Figure 1E), *Breviolum* and *Fugacium* form a sister clade to *Cladocopium*, a trend observed in our reference tree (Figure 1G). This variation suggests that differential evolutionary pressures act on these distinct genomic regions. Although taxa of *Breviolum* and *Cladocopium* are known to be symbiotic, whether *F. kawagutii* is symbiotic or free-living remains to be clarified (Suggett et al., 2015; Saad et al., 2022). The limited representation of these genera in our data here is inadequate for investigating correlation of their phylogeny to lifestyle; data from more taxa would help clarify this.

Within *Symbiodinium*, *S. pilosum* and *S. natans* are free-living whereas the others form symbioses. Our LSU rRNA reference tree indicates that *S. pilosum* is the most anciently diverged lineage within this genus, followed by *S. natans*, and subsequently the symbiotic lineages; this is also observed in the earlier LSU rRNA tree (LaJeunesse et al., 2018) and our tree inferred from protein sequences (Figure 1D). The trees inferred from the rmWGS (Figure 1B) and CDS (Figure 1C) data sets, however, indicate divergence of *S. pilosum* after the split of *S. natans* instead (i.e. and *γ* in the figures). This observation is not surprising because the clade excluding *S. pilosum* is not robustly supported (bootstrap support = 37% in Figure 1B and 68% in the tree in LaJeunesse et al. (2018), suggesting a subtly different phylogenetic signal in the CDS and rmWGS regions compared to protein sequences and the data used to infer the reference tree. In general, we observe species clades that reflect lifestyles in the protein-coding region-based trees, for example, the clear separation of free-living *S. natans* and *S. pilosum* from the other symbiotic *Symbiodinium* taxa. This result was not observed in the intron and repeat-based trees (Figures 1E,F). Interestingly, the WGS (Figure 1A) and intron (Figure 1F) trees indicate the divergence of symbiotic *S. tridacnidorum* (*α* in the figures) prior to the split of the two free-living species and other symbiotic *Symbiodinium*, whereas the *S. tridacnidorum* clade (*α*) interrupts the two free-living species (*β* and *γ*) in the tree inferred from repeats (Figure 1E). Given that repeats comprise a substantial percentage (20%–40% in Symbiodiniaceae, 69%–70% in outgroup *Polarella*; Supplementary Table 1) of these four assembled genomes, the differential G + C content in the annotated repeats in these genomes may have contributed to our observations in the *k*-mer based trees (Supplementary Figure 5). The mean G + C% of repeats in the genome of *S. natans* (49.19%) is similar to that in *S. tridacnidorum* CCMP2592 (48.34%) and that of *S. pilosum* (45.13%) is similar to that in *S. tridacnidorum* Sh18 (45.48%). A difference of 3% G + C can contribute 29% of parsimoniously informative sites in an alignment (Gruber et al., 2007), therefore potentially biasing tree inference. In multiple sequence alignment, sequences of extreme G + C (i.e. low-complexity) are problematic as the biological significance of these aligned regions is not readily discernible (or is simply ignored); this complication does not extend to the *k*-mer-based phylogenetic method that bypasses the alignment step.

The variable positions of the three *Cladocopium* taxa among the trees in Figure 1 may be attributed to the incomplete genome data of the C15 isolate that were generated from *in hospite* coral tissue (Robbins et al., 2019), instead of cultured cells as for all the other genomes. Even so, the position of the monophyletic clade of *Cladocopium* is not in dispute and is robustly supported in our reference (Figure 1G; bootstrap support = 98%) and the earlier phylogeny (bootstrap support = 100%; LaJeunesse et al., 2018).

### Repeats and Non-genic Regions Are More Evolutionarily Conserved Than Genic Regions

**Figure 2:**
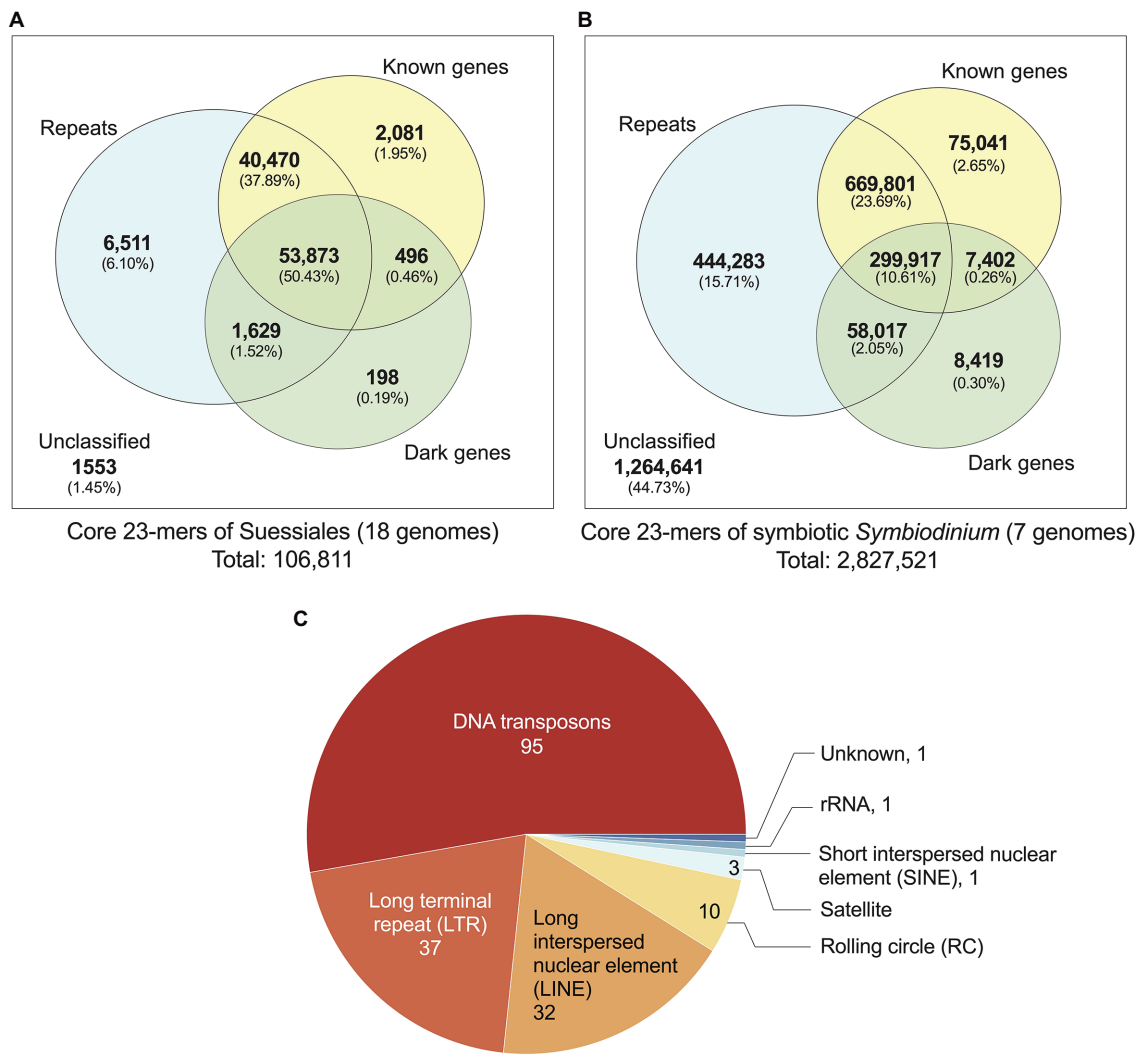
Annotated genome features associated with core 23-mers identified for: **(A)** all 18 genomes of Suessiales, and **(B)** the seven genomes of symbiotic *Symbiodinium*. The Venn diagrams shown in **(A)** and **(B)** are not to scale. **(C)** Repeat classes identified as significantly more abundant and/or significantly more conserved in symbiotic *Symbiodinium* than in free-living members of this genus.

We identified 106,811 distinct core *k*-mers (at *k* = 23) for all 18 dinoflagellate genomes and linked them to annotated genome features (see Materials and Methods). Conservation of these *k*-mers in all 18 genomes reflects their evolutionary importance in Suessiales that includes the family Symbiodiniaceae. Of the 106,811 core 23-mers, 105,258 (98.55%) are present in one or more annotated genome features: 102,488 mapped to annotated repeats [95,972 (89.84% of 106,811) mapped to regions of known and/or dark genes] and 2,081 mapped exclusively to regions of known genes (Figure 2A). The greater prevalence of core *k*-mers (6,511; 6.10%) exclusive in the repetitive elements and non-protein-coding regions compared to those (2,775; 2.60%) exclusive in genic regions (i.e. regions of protein-coding genes inclusive of introns and exons) indicates that non-genic regions are more evolutionarily conserved than genic regions in Suessiales genomes, lending support to their extensive genomic divergence (González-Pech et al., 2021). Supplementary Figure 6 presents a phylogenetic tree in which each node is annotated with the number of *k*-mers shared by the taxa encompassed by the node, in the context of evolutionary time. These shared *k*-mers represent defining features conserved across each clade contained by the node.

Closely related taxa in general are expected to share a larger number of core *k*-mers than more-distantly related taxa (Supplementary Figure 6). To correlate core *k*-mers to symbiotic lifestyle, we identified 2,827,521 core 23-mers in symbiotic *Symbiodinium* (seven genomes; see Materials and Methods). The higher abundance of core *k*-mers in these taxa likely reflect their more-recent divergence [estimated ~4.8 million year ago (MYA)] compared to the 125,958 core *k*-mers for Symbiodiniaceae (estimated divergence ~165.9 MYA; Supplementary Figure 6). The identified genome features that encompass these core 23-mers (Figure 2B) are comparable to what we observed for Suessiales, with a smaller proportion (1,562,880; 55.27%) recovered in one or more annotated features, and a larger proportion (444,283; 15.71%) exclusively recovered in annotated repeats. A total of 90,862 core 23-mers (3.21%) mapped only to regions of known and/or dark genes (75,041 only in known genes, 8,419 only in dark genes, 7,402 in both; Figure 2B). This result reveals a greater extent of conserved *k*-mers in genic regions among the seven genomes of symbiotic *Symbiodinium* than in all 18 genomes of Suessiales. In addition, some regions of repetitive elements and non-protein-coding regions remain evolutionarily conserved, and this trend is likely common to dinoflagellate genomes.

### Repeats Significant in Symbiotic Versus Free-Living Symbiodinium

The transition from a free-living to a symbiotic lifestyle is thought to be marked by a phase of genome instability, structural rearrangement and burst of mobile genetic element activity (González-Pech et al., 2019). We assessed if the annotated repeats in the genomes of seven symbiotic *Symbiodinium* are significantly different to those found in the genomes of free-living *Symbiodinium* (i.e. *S. natans* and *S. pilosum*). We assessed the repeats in two ways: (a) their abundance based on proportional length relative to total assembled genome sequences and (b) their sequence conservation based on Kimura divergence of known repeat families (Supplementary Table 2). Of the 825 distinct repeat types annotated in all seven genomes, 180 (21.8%) encompassing eight classes (including the ‘Unknown’ class) were found to be significantly overrepresented in proportion (159), significantly more conserved based on Kimura distances (20) relative to known repeat sequences, or both (1); the Dp_Skipper-1-I (LTR/Gypsy class) is significant in both tests (*p* ~ 0.04 in both cases; Supplementary Table 2). This result suggests that repeat content in the genomes of symbiotic lineages is significantly different from that in those of free-living lineages, which is largely explained by repeat types that occupy significantly larger proportions of the genomes in symbiotic lineages, and to a lesser extent, repeat types that are more evolutionarily conserved. Of the 180 repeat types, the majority (52.8%) are from the DNA class, followed by long-terminal repeat (LTR; 20.6%) and long interspersed nuclear element (LINE; 17.8%) classes. DNA transposons and LTRs occur in two- to three-fold greater abundance in the genome of symbiotic *S. tridacnidorum* relative to the genome of free-living *S. natans* (González-Pech et al., 2021). Sequences in these repeat classes are divergent in *Symbiodinium* genomes (i.e. Kimura distances centred between 15 and 40; González-Pech et al., 2021); we cannot dismiss that some may represent non-functional relics that are retained in the genome sequences. However, the greater abundance of transposable elements: for example, copia and gypsy retrotransposons, the DNA transposons of mariners, and the rolling-circle transposons of helitrons we observed in genomes of the seven symbiotic *Symbiodinium* (Supplementary Table 2) lends support to the earlier results of Gonzalez-Pech et al. (2021) and to the notion that enhanced activity and/or expansion of transposable elements is associated with symbiotic lineages, contributing to their dynamic genome evolution (Lin et al., 2015; Song et al., 2017; González-Pech et al., 2019).

### Representing Alignment-Free Phylogenies as Networks

**Figure 3:**
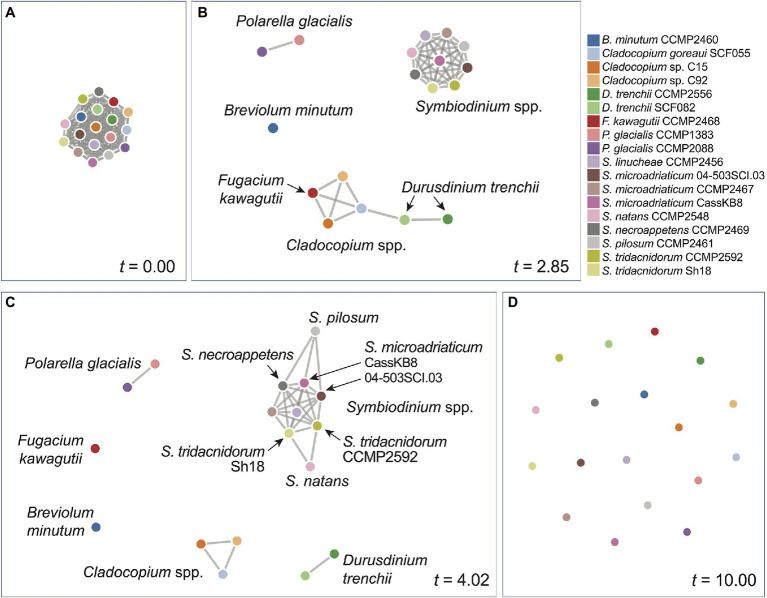
Network of genome relatedness of 18 Suessiales inferred using the rmWGS data at different *t*-values: **(A)** *t* = 0.00, **(B)** *t* = 2.85, **(C)** *t* = 4.02, and **(D)** *t* = 10.0. The colour used to represent each genome is shown in the legend.

We inferred a network of relatedness from the distinct genome sequence regions based on *k*-mers (Supplementary Data 2). These networks allow for dynamic visualisation based on the similarity threshold *t* relative to the similarity measure *S* between a pair of genomes (see Materials and Methods), in which any genome pair (i.e. a pair of nodes) with *S ≥ t* is connected with an edge. At the most lenient threshold (*t* = 0), all genomes (i.e. nodes) are interconnected with edges (i.e. a clique), whereas at the most stringent threshold (*t* = 10), all genomes are disconnected. Figure 3 shows the network based on rmWGS data at *t* = 0.00 (Figure 3A), *t* = 2.85 (Figure 3B), *t* = 4.02 (Figure 3C) and *t* = 10.00 (Figure 3D). At *t* = 2.85, a clear separation of the outgroup *P. glacialis*, the genus of *Symbiodinium* (in a clique), *Breviolum*, and the other genera (*Cladocopium* and *Fugacium*) was observed. At *t* = 4.02 (Figure 3C), all distinct genera are separated. In the corresponding rmWGS tree (Figure 1B), the branching order of free-living *S. natans* and *S. pilosum* is different from that in the reference LSU tree (Figure 1G), and they remain more closely related than each of them is to the other *Symbiodinium* taxa. In the network at *t* = 4.02 (Figure 3B), these two species are connected to other symbiotic *Symbiodinium* taxa instead of each other: that is, *S. natans* is connected to two isolates of *S. tridacnidorum*, whereas *S. pilosum* is connected to *S. necroappetens* and two isolates of *S. microadriaticum*. This observation suggests that the distinction between free-living and symbiotic lifestyles is not the only factor contributing to the divergence of Symbiodiniaceae genomes and that the same information based on 23-mers when presented as a network yields additional insights into the evolution of the two free-living *Symbiodinium* species when compared to the tree representation. By not assuming a strict tree-like structure of evolutionary history, the network representation of relatedness captures the signal of vertical and horizontal inheritance, as well as other evolutionary processes that underpin genome evolution of Symbiodiniaceae. The networks we generated (Supplementary Data 2) provide a flexible, dynamic view of genome relatedness among the 18 taxa inferred from distinct genomic regions and across the similarity threshold, thus enabling generation of new hypotheses to drive future research.

### Alignment-Free Phylogenetics in the Genomic Era

The conflicts of phylogenetic signals we observed among distinct genomic sequence regions demonstrate how differential evolutionary pressure acting on these regions has shaped their evolution. By disregarding repeats in whole-genome sequences, the inferred tree is similar to the existing phylogeny based on LSU rRNAs. Repeats constitute, on average, 34% of assembled sequences of each Suessiales genome (Supplementary Table 1); and these estimates remain conservative due to the varying extent of sequence contiguity of the various genome assemblies (Table 1). Even when all protein-coding genes (or the associated proteins) are used in *k*-mer based phylogenetic inference, the phylogenetic signal represents an average of only 7% of the assembled genomes (Supplementary Table 1). Therefore, a key question remains to be resolved: is a tree inferred from genomic regions restricted to marker gene(s) or genic regions an appropriate depiction of the evolutionary history of Symbiodiniaceae taxa? We think this to be an academic argument as described below and that ongoing discussion about the lumping or splitting lineages that are morphologically simple should be adjudicated in the ‘court’ of genome evolution. We are far from having a full understanding of Symbiodinaceae or other microbial eukaryotes to do this as of yet, but progress is rapid in this area and we ignore the majority genome at our peril, when aiming to understand how evolution works.

By not assuming an explicit substitution model of molecular evolution, the applicability and intuitive basis of *k*-mers in alignment-free phylogenetic approaches has been investigated and widely discussed (Posada, 2013; Ragan and Chan, 2013). These methods are sensitive to the quality and divergence of sequence data, as expected when using the conventional alignment-based phylogenetic approach. In addition, the optimal *k* length varies among different data sets and needs to be determined empirically, as we did in this study. Overall, *k*-mer-based phylogenetic approaches have proven robust against among-site rate variation issues and various molecular evolution scenarios based on analysis of simulated and empirical sequence data (Chan et al., 2014; Bernard et al., 2016a). Their demonstrated scalability (Bernard et al., 2016b; Bernard et al., 2018; Jacobus et al., 2021) enables the capture of comprehensive phylogenetic signal from large, whole-genome sequence data. This signal provides useful evidence to guide the taxonomic analysis of microbial eukaryotes, including Symbiodiniaceae (González-Pech et al., 2021; Dougan et al., 2022), for which classification may be confounded by subtle variation in morphology and the ineffectiveness of established phylogenetic markers.

## Conclusion

Our results demonstrate the utility of alignment-free phylogenetic methods based on *k*-mers to efficiently infer evolutionary history from the massive whole-genome sequence data sets of dinoflagellates. Sequence regions implicated in protein coding (i.e. CDS and the coded protein sequences) exhibit phylogenetic signal that is largely consistent with the phylogeny inferred from multiple sequence alignment of selected marker genes (e.g. LSU rRNA), but sequences of introns and repeats, which constitute a large proportion of the genomes and are more evolutionarily conserved, exhibit a different signal that appears to impact phylogenetic inference from whole-genome sequences. Introns are conserved despite having a higher evolutionary rate than exons (Lynch, 2002; Rogozin et al., 2003; Parenteau and Abou Elela, 2019). Although repeats more readily accumulate mutations due to neutral evolution or slippage during DNA replication, our results reveal over 50% of identified core *k*-mers of Suessiales (and of the symbiotic *Symbiodinium*) taxa are implicated in the annotated repeat regions, indicating genome-wide conservation of these elements. In addition, simple repeats characterised as tandemly repeated short sequences may enhance genetic variation while exerting minimal load on the host (Kashi and King, 2006). Therefore, genomic diversity of these dinoflagellates is contributed in part by interesting sequence conservation patterns in introns and in repeat content.

Whole-genome data enable the analysis of ‘total’ phylogenetic signal that has resulted from complex evolutionary processes underpinning the evolution of Symbiodiniaceae. In the absence of whole-genome data, selected phylogenetic markers, such as LSU rRNAs, a curated set of ‘metabarcode’ genes, or strictly (single-copy) orthologous genes remain appropriate and relevant for inferring a representative phylogenetic relationship. However, the increasing amount of whole-genome data offer a more comprehensive accounting of molecular evolution and allow testing of new hypotheses about lineage diversification and niche specialisation in Symbiodiniaceae and other taxa (Dougan et al., 2022). We argue that the best use of genome data to understand evolutionary processes is to use all of the data and that it is counter-intuitive to ignore the large proportion of genome sequences that are non-protein-coding and/or repetitive. In this regard, alignment-free phylogenetic methods (e.g. based on *k*-mers), while not relying on sophisticated evolutionary models, provide a scalable approach to infer biologically meaningful evolutionary relationships from whole-genome data. Support for the inferred clades on an alignment-free tree can be assessed using the jackknife technique (Bernard et al., 2016a; Jacobus et al., 2021) that provides a value similar to bootstrap support in a maximum-likelihood tree. What the different sequence regions in Symbiodiniaceae genomes can teach us about adaptation and evolutionary processes remain to be more thoroughly investigated in future studies as done for model taxa, such as *Drosophila* (Oti et al., 2018), among others (Gemmell, 2021).

## Data Availability Statement

The original contributions presented in the study are included in the article/Supplementary Material, and further inquiries can be directed to the corresponding authors.

## Author Contributions

RL, KD, and CC conceived the study and designed the research. RL, KD, YC, and SS conducted the research and performed all computational analyses. RL prepared the first draft of the manuscript. KD, DB, and CC supervised the research. DB and CC contributed to writing and iterative revisions of the manuscript. All authors contributed to the article and approved the submitted version.

## Funding

This work is supported by an Australian Research Council grant (DP190102474 awarded to CC and DB). DB was also supported by a research grant from the National Aeronautics and Space Administration (NASA; 80NSSC19K0462) and a NIFA-USDA Hatch grant (NJ01180).

## Conflict of Interest

The authors declare that the research was conducted in the absence of any commercial or financial relationships that could be construed as a potential conflict of interest.

## Publisher’s Note

All claims expressed in this article are solely those of the authors and do not necessarily represent those of their affiliated organizations, or those of the publisher, the editors and the reviewers. Any product that may be evaluated in this article, or claim that may be made by its manufacturer, is not guaranteed or endorsed by the publisher.

## Footnotes

^1^ http://www.repeatmasker.org/

^2^ https://d3js.org/
