# Supplementary material for: Alignment-Free Analysis of Whole-Genome Sequences From Symbiodiniaceae Reveals Different Phylogenetic Signals in Distinct Regions
Source: Front Plant Sci. 2022 Apr 26;13:815714. doi: 10.3389/fpls.2022.815714 (PMC9087856; doi:10.3389/fpls.2022.815714)
Supplement: Supplementary file 2 [file Data_Sheet_2.ZIP › repeat_51mer_network.html]

Repeats - 51-mer network


Repeats - 51-mer network


### Threshold (min 0)(max 10) selected :

Search
