# Supplementary material for: Alignment-Free Analysis of Whole-Genome Sequences From Symbiodiniaceae Reveals Different Phylogenetic Signals in Distinct Regions
Source: Front Plant Sci. 2022 Apr 26;13:815714. doi: 10.3389/fpls.2022.815714 (PMC9087856; doi:10.3389/fpls.2022.815714)
Supplement: Supplementary file 3 [file Data_Sheet_3.PDF]

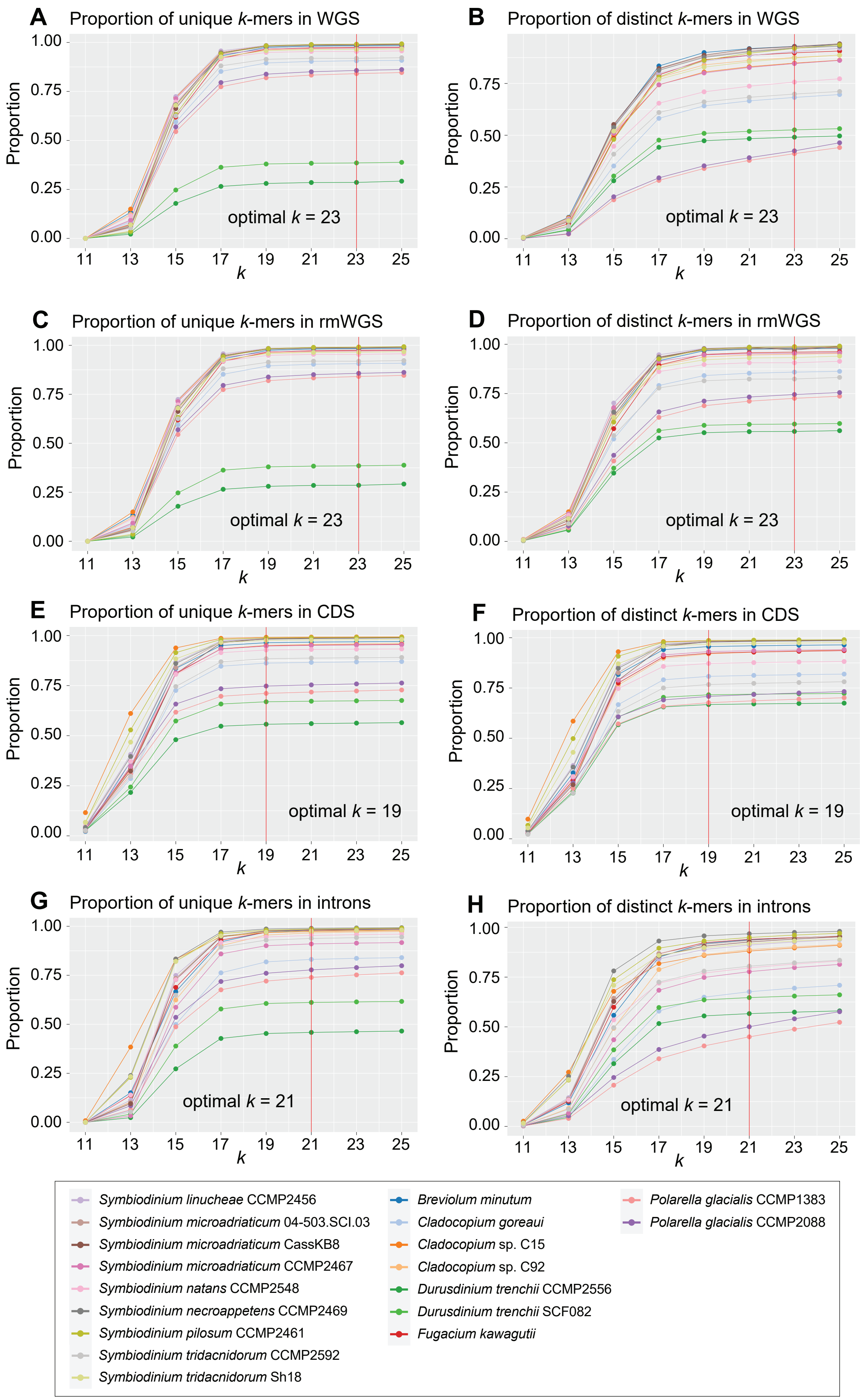

Supplementary Figure 1. The proportion of unique *k*-mers and the proportion of distinct *k*-mers for the datasets of WGS, rmWGS, CDS and introns, with red line on each graph indicates the chosen optimal *k*.
