# Supplementary material for: Alignment-Free Analysis of Whole-Genome Sequences From Symbiodiniaceae Reveals Different Phylogenetic Signals in Distinct Regions
Source: Front Plant Sci. 2022 Apr 26;13:815714. doi: 10.3389/fpls.2022.815714 (PMC9087856; doi:10.3389/fpls.2022.815714)
Supplement: Supplementary file 4 [file Data_Sheet_4.PDF]

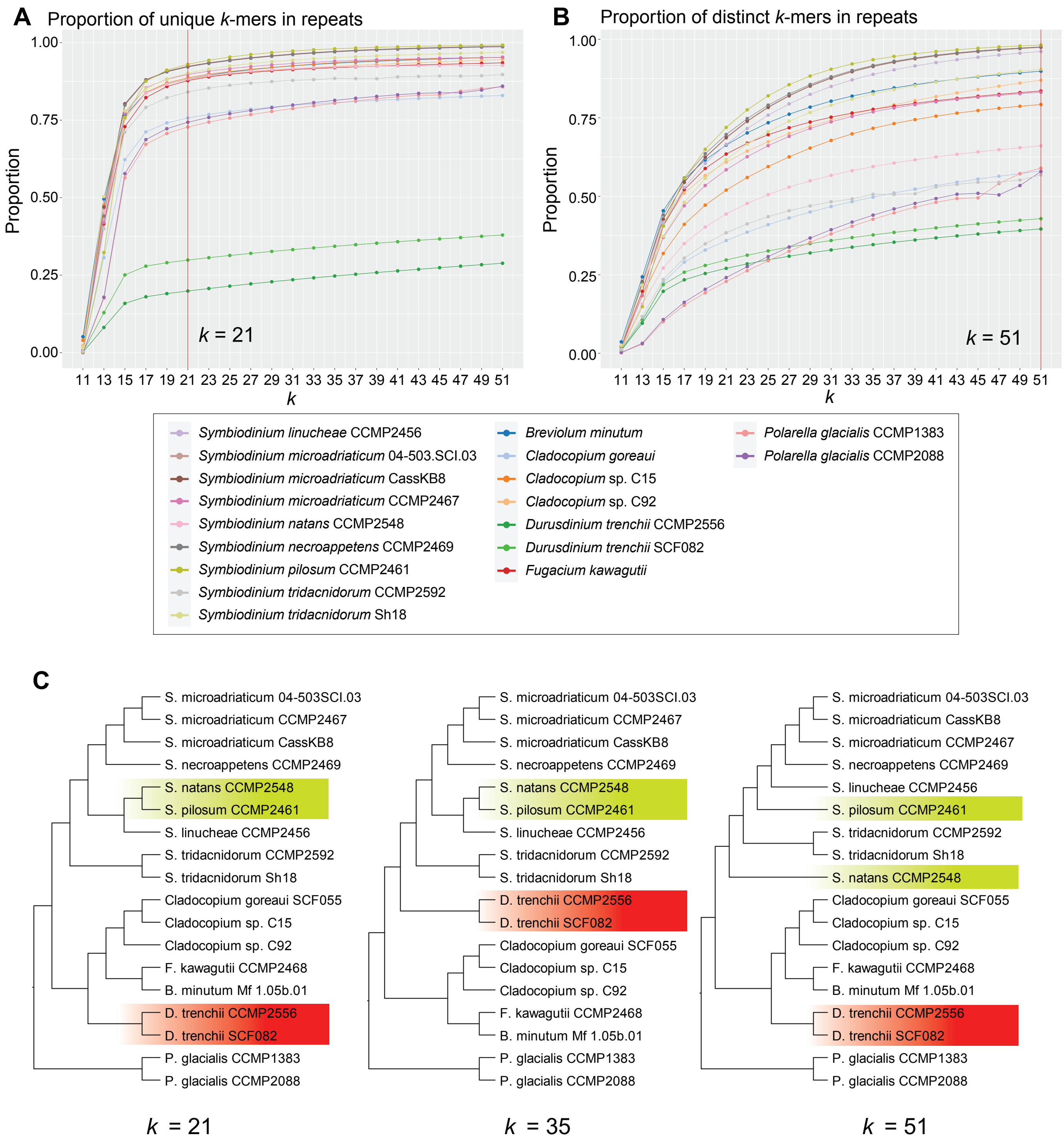

Supplementary Figure 2. The proportion of (A) unique *k*-mers and of (B) distinct *k*-mers for the dataset of repeats, and (C) phylogenetic trees inferred from this dataset independently at *k* = 21, 35 and 51.
