# Supplementary material for: Alignment-Free Analysis of Whole-Genome Sequences From Symbiodiniaceae Reveals Different Phylogenetic Signals in Distinct Regions
Source: Front Plant Sci. 2022 Apr 26;13:815714. doi: 10.3389/fpls.2022.815714 (PMC9087856; doi:10.3389/fpls.2022.815714)
Supplement: Supplementary file 5 [file Data_Sheet_5.PDF]

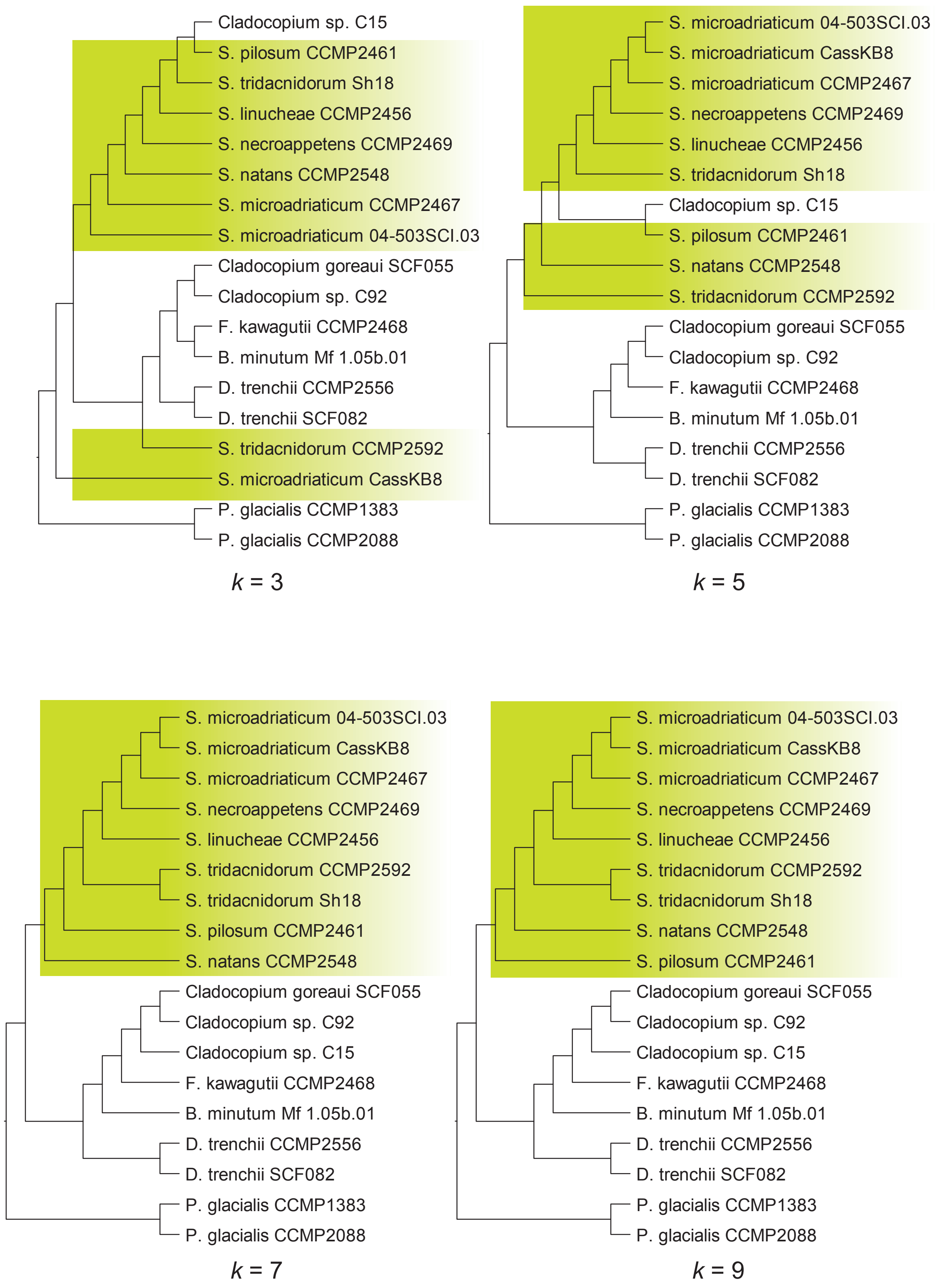

Supplementary Figure 3. Phylogenetic trees inferred from the protein dataset independently at k = 3, 5, 7 and 9, with *Symbiodinium* taxa highlighted on the trees.
