# Supplementary material for: Alignment-Free Analysis of Whole-Genome Sequences From Symbiodiniaceae Reveals Different Phylogenetic Signals in Distinct Regions
Source: Front Plant Sci. 2022 Apr 26;13:815714. doi: 10.3389/fpls.2022.815714 (PMC9087856; doi:10.3389/fpls.2022.815714)
Supplement: Supplementary file 6 [file Data_Sheet_6.PDF]

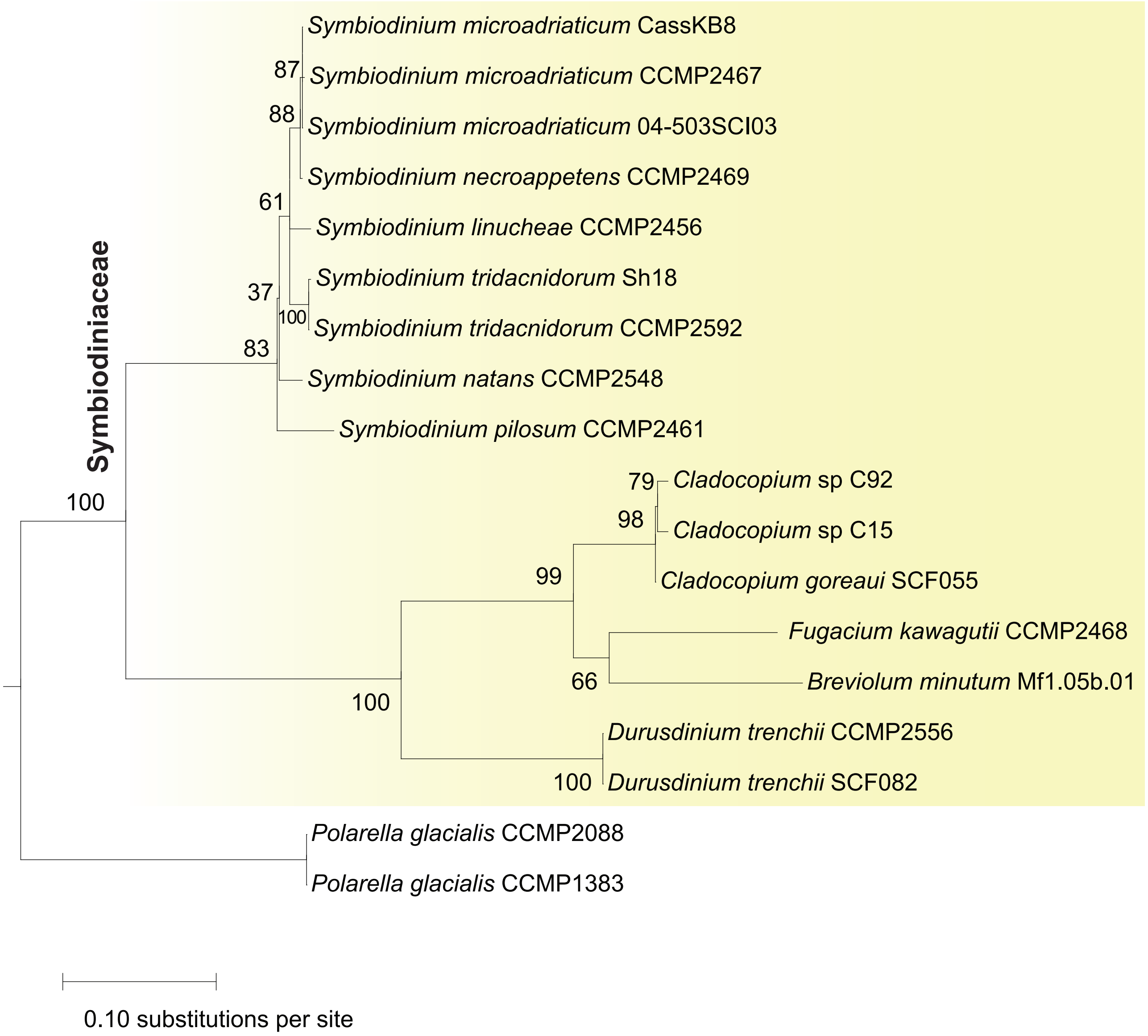

Supplementary Figure 4. The maximum-likelihood reference tree inferred from multiple sequence alignment of LSU rRNA (Supplementary Data 1), showing ultrafast bootstrap support (from 1000 sample replicates). Unit of branch length is number of substitutions per site.
