# Supplementary material for: Alignment-Free Analysis of Whole-Genome Sequences From Symbiodiniaceae Reveals Different Phylogenetic Signals in Distinct Regions
Source: Front Plant Sci. 2022 Apr 26;13:815714. doi: 10.3389/fpls.2022.815714 (PMC9087856; doi:10.3389/fpls.2022.815714)
Supplement: Supplementary file 7 [file Data_Sheet_7.PDF]

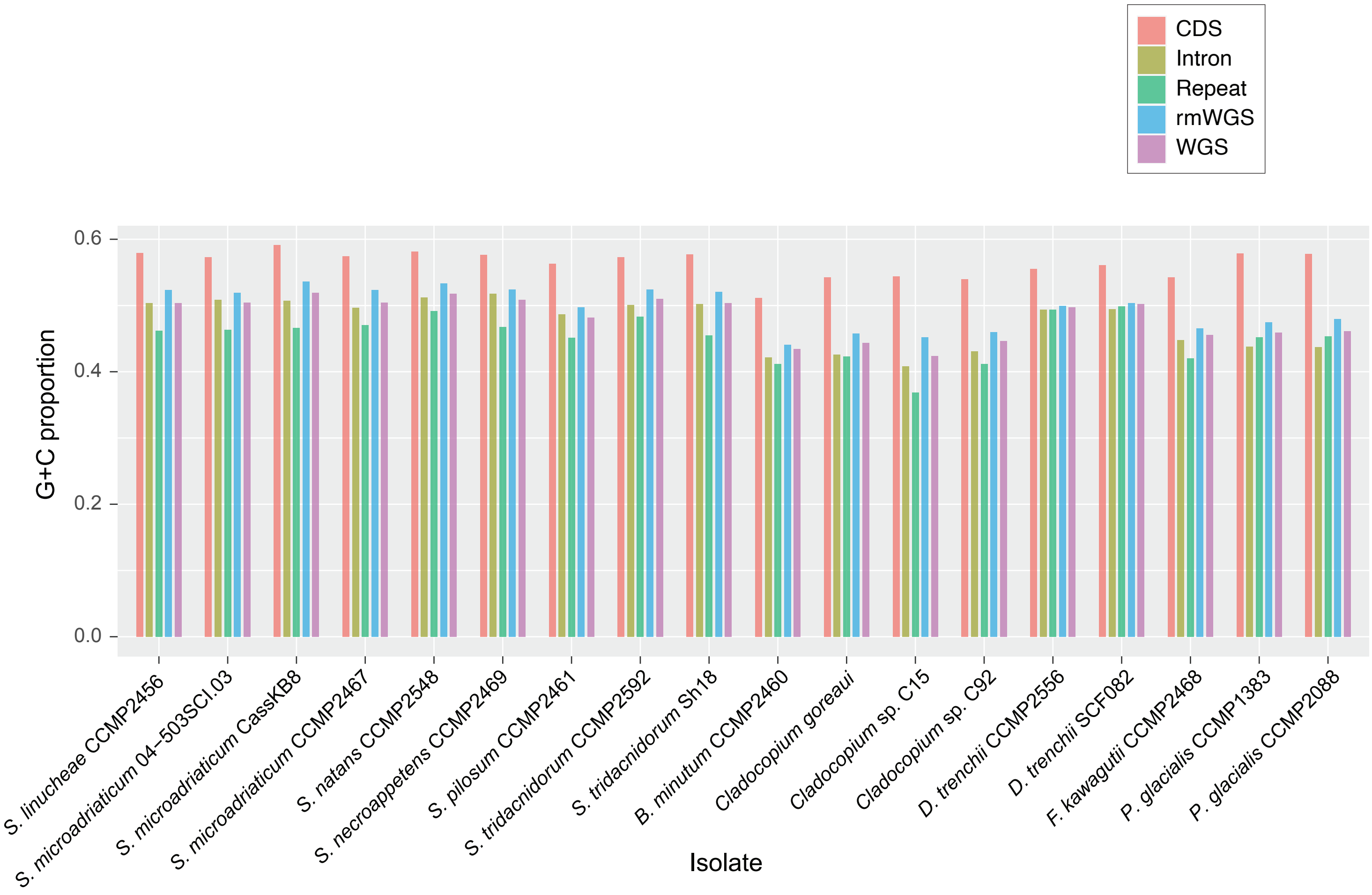

Supplementary Figure 5. G+C proportion for each nucleotide dataset (CDS, introns, repeats, rmWGS and WGS) used in this study, for each of the 18 taxa.
