# Supplementary material for: Alignment-Free Analysis of Whole-Genome Sequences From Symbiodiniaceae Reveals Different Phylogenetic Signals in Distinct Regions
Source: Front Plant Sci. 2022 Apr 26;13:815714. doi: 10.3389/fpls.2022.815714 (PMC9087856; doi:10.3389/fpls.2022.815714)
Supplement: Supplementary file 8 [file Data_Sheet_8.PDF]

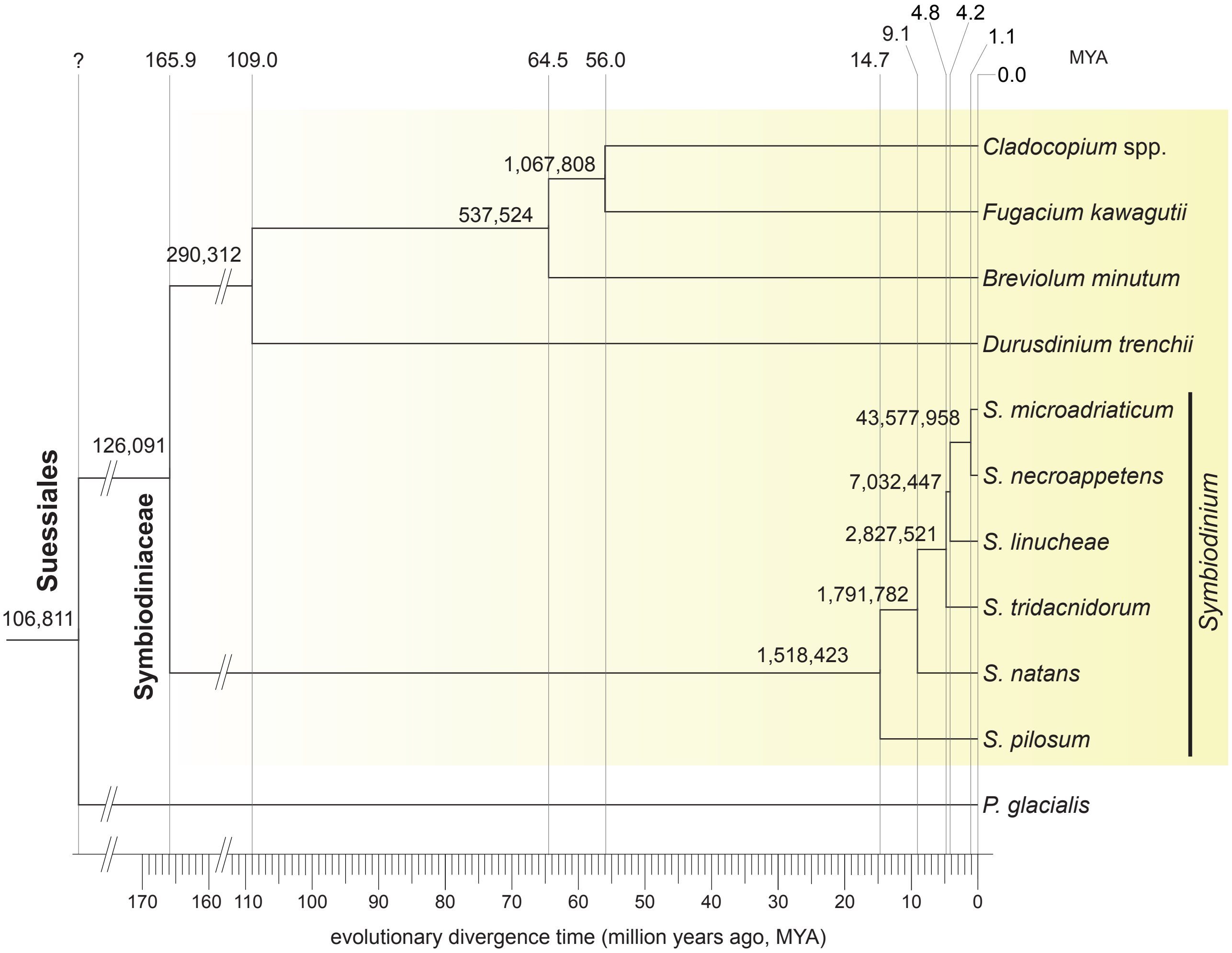

Supplementary Figure 6. Number of core 23-mers recovered at each node of the Suessiales tree, including estimated evolutionary divergence times of distinct clades based on LaJeunesse et al. (2018).
